# Health Tract, No. 180

**Published:** 1863-12

**Authors:** 


HEALTH TRACT, No. 180.

### CHILDREN'S FEET.

LIFE-LONG discomfort, disease, and sudden death often come to children through the inattention, ignorance, or carelessness of the parents. A child should never be allowed to go to sleep with cold feet; the thing to be last attended to, in putting a child to bed, should be to see that the feet are dry and warm; neglect of this has often resulted in a dangerous attack of croup, diphtheria, or fatal sore throat.

Always, on coming from school, on entering the house from a visit or errand in rainy, muddy, or thawy weather, the child's shoes should be removed, and the mother should herself ascertain if the stockings are the least damp; and if so, should require them to be taken off, the feet held before the fire and rubbed with the hand until perfectly dry, and another pair of stockings be put on and another pair of shoes, while the other stockings and shoes should be placed where they can be well dried, so as to be ready for future use at a moment's notice.

There are children not ten years of age suffering with corns from too close-fitting shoes, by the parent having been tempted to "take" them because a few cents were deducted from the price, while the child's foot is constantly growing. A shoe large enough with thin stockings is too small on the approach of cold weather and thicker hose, but the consideration that they are only half worn is sufficient sometimes to require them to be worn, with the result of a corn, which is to be more or less of a trouble for fifty years perhaps; and all this to save the price of a pair of half-worn shoes! No child should be fitted with shoes without putting on two pair of thick woolen stockings, and the shoe should go on moderately easy even over these. Have broad heels, and less than half an inch in thickness.

Tight shoes inevitably arrest the free circulation of the blood and nervous influences through the feet, and directly tend to cause cold feet; and health with habitually cold feet is an impossibility.

That parent is guilty of a criminal negligence who does not always see to it that each child enters the church and school-house door with feet comfortably dry and warm. Grown persons of very limited intelligence know that, as to themselves, damp feet endanger health and life, however robust; much more so must it be to the tender constitution of a growing child.

I have never known a shoemaker, whether in sending home a pair of new shoes or old ones repaired, to fail leaving several pegs or iron nails to project through the sole on the inside. The result is, that often in a single day, the excitement of play preventing a child from noticing any discomfort, the stockings are cut through in several places and ugly sores are made in the soles of the feet, to be an annoyance and a trouble for a week afterward; beside the unnecessary work given to an already overtasked mother in mending the stockings. To avoid the results of such inexcusable neglect, and also to make it more sure that pegs and nails should not "work through" by the shrinkage of the leather, and also to keep the feet dry, there should be worn between the leather of the shoe and the stocking a piece of cork, or soft, thick pasteboard, lined at the bottom with a piece of oiled silk, and on the upper-side touching the stocking the lining should be of Canton flannel; each person should have two pair of these, to be worn on alternate days.

